# Transcriptional Responses of *Fusarium graminearum* Interacted with Soybean to Cause Root Rot

**DOI:** 10.3390/jof7060422

**Published:** 2021-05-27

**Authors:** Muhammd Naeem, Maira Munir, Hongju Li, Muhammad Ali Raza, Chun Song, Xiaoling Wu, Gulshan Irshad, Muhammad Hyder Bin Khalid, Wenyu Yang, Xiaoli Chang

**Affiliations:** 1 College of Agronomy & Sichuan Engineering Research Center for Crop Strip Intercropping System, Sichuan Agricultural University, Chengdu 611130, China; muhammdnaeem201@gmail.com (M.N.); mairamunir23@gmail.com (M.M.); lihongjucomeon@163.com (H.L.); razaali0784@yahoo.com (M.A.R.); songchun@sicau.edu.cn (C.S.); wulx@sicau.edu.cn (X.W.); haider2323@gmail.com (M.H.B.K.); mssiyangwy@sicau.edu.cn (W.Y.); 2 Department of Plant Pathology, PMAS Arid Agriculture University, Rawalpindi 46000, Pakistan; gulshanirshadpp@gmail.com

**Keywords:** *Fusarium* *graminearum*, differentially expressed genes, carbon metabolism, ribosome, peroxisomes pathway, maize-soybean strip relay intercropping

## Abstract

*Fusarium graminearum* is the most devastating pathogen of *Fusarium* head blight of cereals, stalk and ear of maize, and it has recently become a potential threat for soybean as maize-soybean strip relay intercropping is widely practiced in China. To elucidate the pathogenesis mechanism of *F. graminearum* on intercropped soybean which causes root rot, transcriptional profiling of *F. graminearum* at 12, 24, and 48 h post-inoculation (hpi) on soybean hypocotyl tissues was conducted. In total, 2313 differentially expressed genes (DEGs) of *F. graminearum* were annotated by both KEGG pathway and Gene Ontology (GO) analysis. Among them, 128 DEGs were commonly expressed at three inoculation time points while the maximum DEGs were induced at 24 hpi. In addition, DEGs were also rich in carbon metabolism, ribosome and peroxisome pathways which might contribute to carbon source utilization, sexual reproduction, virulence and survival of *F. graminearum* when infected on soybean. Hence, this study will provide some basis for the deep understanding the pathogenesis mechanism of *F. graminearum* on different hosts and its effective control in maize-soybean strip relay intercropping systems.

## 1. Introduction

Soybean (*Glycine max* L.) is an economically important oilseed crop worldwide and is popular as a source of edible and supplementary nutritious food products [1]. Particularly in the southwestern areas of China, the combination of legume (soybean) and cereal (maize) under relay strip intercropping exhibits high intercrop yield and produces high protein soybeans because of the improved land equivalent ratio and solar-thermal resources [2,3,4]. Currently, this system is being widely practiced in Southwest China and other single-season cropping areas [3]. However, soybean root rot caused by several phytopathogenic fungi, mainly *Fusarium* species, has been frequently reported as the continuous cropping is practiced widely [5,6,7,8,9]. The outbreaks of *Fusarium* diseases prevailed in soybean when cultivated in rotation with sorghum, corn, wheat, and other grass crops.

*Fusarium graminearum*, anamorph of *Gibbriella zeae*, is the most prevalent pathogen to cause *Fusarium* head blight (FHB) in small cereals [10,11] as well as ear and stalk rot of maize [12,13], and it has also been frequently isolated from soybean rotted roots and seeds in the USA, Argentina, and China [5,14,15,16]. Previous studies demonstrated that in the soybean-wheat rotation cropping, the residues of soybean stem and root after harvesting were easily colonized by *F. graminearum* and thus used as primary inoculum of the subsequent crop wheat [17]. Despite the severe yield reduction, *F. graminearum* can contaminate cereal grains by producing several mycotoxins such as zearalenone (ZEA) and deoxynivalenol (DON), which have hazardous effects on human as well as animal health [18,19]. Due to the lack of effective resistance resources, several agronomic practices and fungicide treatments are often conducted to prevent the *F. graminearum* infection during epidemic years [20]. The understanding of the infection process and pathogenesis mechanism of *F. graminearum* in the interaction with different hosts is helpful for sustainable and effective management of *F. graminearum*-related diseases.

In recent decades, extensive work has been carried out to study genetic evolution, population biology, infection and pathogenesis mechanisms of *F. graminearum* [12,21]. Many advances in Affymetrix gene chips, sequencing techniques, and bio-information methods help to explore the genome and transcriptome of *F. graminearum* when interacting with different host plants [16,17,18]. Since the genome of *F. graminearum* was released in 2007 [21], the genomic data of more than 64 *Fusarium* species has been published on a fungal transcription factor database (FTFD, http://ftfd.snu.ac.kr/, accessed on 20 May 2021) [22,23]. Recently, the stage-specific fungal gene profiling in planta has elucidated the molecular strategies of *F. graminearum* when infected on wheat coleoptiles [24]. In addition, a transcriptional analysis of *F. graminearum* in response to a fungicide azole of *Fusarium* head blight of cereals was reported [25]. Several signaling pathways have previously been explored and function in the plant infection, pathogenicity and condition of many fungi [26,27,28]; however, numerous works are still needed to unveil the pathogenesis-related signaling pathways and key regulation genes involved in the infection processes of different hosts.

Currently, the severe damage of soybean root rot has appeared in the maize-soybean relay strip intercropping in China, but the infection and pathogenesis mechanism of *F. graminearum* on soybean have not been clearly elucidated. Herein, the transcriptional profiling of *F. graminearum* when infected on soybean was analyzed and the important signaling pathways responsible for its infection and pathogenicity were explored. This study will provide insights for the detailed molecular pathogenesis mechanism of *F. graminearum* on soybean and could be useful in designing sustainable management strategies of soybean root rot during maize-soybean relay strip cropping.

## 2. Materials and Methods

### 2.1. Plant Preparation and Growth Conditions

Soybean cultivar Nandou12, moderately susceptible to *Fusarium* species, has widely been used in maize-soybean relay strip intercropping in Southwest China. In this study, seeds of Nandou12 were washed with tap water for 15 min and germinated for 2 days at room temperature in the dark. Germinated seeds were then planted in Plantmax nutrient soil at 25 °C for 5 days with 16 h light alternated with 8 h dark in a controlled growth chamber (GXM illumination cultivation cabinet, Ningbo Jiangnan Instrument Factory, Ningbo, China) until the first compound leaf appeared.

### 2.2. Preparation and Inoculation of the Pathogen

*Fusarium graminearum* (isolate *F.g_106*) was isolated from the diseased root of field-grown soybean and previously identified as the pathogenic species of soybean root rot [5]. The isolate was cultured on PDA (200 g∙L^−1^ potato, 10 g∙L^−1^ glucose anhydrous, and 15 g∙L^−1^ agar) provided with 50 μg∙mL−1 streptomycin and later was incubated at 25 ± 2 °C for 7–10 days in darkness. The hypocotyl of each soybean seedling was pinched by a steel pin, inoculated with a fresh mycelium cake, and then sealed with the Parafilm sealing film (PM-996, Bemis, Neenah, WI, USA). About 1-cm long hypocotyl around the infection sites from each seedling were harvested at 12, 24 and 48 hpi while three mycelium samples of *F. graminearum* isolate were used as negative control. Nine seedling hypocotyls were harvested at each indicated time point and mixed as test samples. Three biological replicates were independently performed, and all collected samples were stored at −80 °C for further analysis.

### 2.3. Extraction of Total RNA

For RNA-seq and qRT-PCR analysis, all hypocotyl samples were ground in the liquid nitrogen using pestle and mortar, and total RNA was extracted using a Trizol extraction kit (Sigma-Aldrich, Shanghai, China) following the producer’s protocol. The extracted RNA was subjected to a DNA-free DNase (Qiagen, Shanghai, China) to remove the contamination of potential genomic DNA and further cleaned by RNeasy mini spin columns (Qiagen, Shanghai, China). The RNA concentrations were estimated using the Qubit 2.0 Fluorometer (Life Technologies, CA, USA). The RNA integrity was checked by the Nano 6000 Assay Kit of the Agilent Bioanalyzer system 2100 (Agilent Technologies, CA, USA).

### 2.4. cDNA Library Construction and Illumina RNA-Sequencing

cDNA libraries were synthesized using NEBNex Ultra TM RNA library Pre-Kit for Illumina (BioLabs, New England, USA) following the manufacturer’s instructions. Poly-T oligo-attached magnetic beads were used to purify the mRNA from total extracted RNA, and fragmentation was performed with divalent cations under high temperature in NEBNext First Strand Synthesis Reaction Buffer. The first strand of cDNA was generated using random hexamer primer according to the protocol of M-MuLV Reverse Transcriptase, and the second strand of cDNA was subsequently synthesized by using DNA Polymerase I and RNase H. The adapter with hairpin loop structure was ligated to prepare for hybridization, and then cDNA fragments with 200–250 bp in length were purified with AMPure XP system (Beckman Coulter, Beverly, MA, USA). After enrichment of size-selected and adaptor-ligated DNA by PCR amplification, cDNA libraries were quantified with Qubit 2.0 and qualified by Agilent Bioanalyzer 2100. The cluster was generated by cBot using TruSeq PE Cluster Kit v4-cBot-HS (llumina, San Diego, CA, USA) and then sequenced on an Illumina Hiseq 2500 platform. The library construction and sequencing were performed at BMK Biotechnology Corporation in Beijing.

### 2.5. RNA-Seq Data Analysis

Raw data reads were cleaned using Cutadapt v1.1 to remove reads containing adapter, reads trimmed to less than 25 bp, and the low-quality reads (Q ≤ 0). All clean reads were compared to the reference genome of *F. graminearum* (https://www.ncbi.nlm.nih.gov/genome/58?genome_assembly_id=284608, accessed on 20 May 2021) using TopHat2 (http://ccb.jhu.edu/software/tophat/index.shtml, accessed on 20 May 2021) [29], and the uniquely mapped reads were identified. The mapped reads were spliced by Cufflinks software (http://cufflinks.cbcb.umd.edu/, accessed on 20 May 2021). Quantification of gene expression level was established by FPKM [30]. Differential expression analysis of three inoculation time intervals was performed using the DESeq R package (1.10.1). Genes with an adjusted False Discovery Rate (FDR) < 0.01 and Fold Change (log2FC ≥ 2) were assigned as differentially expressed. Function annotation and enrichment analysis of genes were performed based on Gene Ontology (GO) [31] and KEGG Orthology (http://www.genome.jp/kegg/, accessed on 20 May 2021) [32]. KOBAS software [33] was used to test the statistical enrichment of differential expression genes (DEGs) in KEGG pathways.

### 2.6. qRT-PCR Verification

For the verification of gene expression, cDNA were performed for qRT-PCR with a 10 µL reaction mixture containing 1 µL of cDNA, 1 µL of each reverse and forward primer, 3 µL of RNAse free water and 5 µL of SYBR green mixture (SYBR green, Toyobo, New York, NY, USA). The cDNA was synthesized using the HiScript II Q Select RT Supermix for qPCR (+gDNA wiper) cDNA synthesis kit according to the manufacturer instructions (Vazyme Biotech, Nanjing, China). qRT-PCR was performed by using a Real time PCR cycler (Eppendorf AG 22331, Hamburg, Germany) with the thermal cycling conditions as follows: 50 ℃ for 2 min, 95 ℃ for 2 min, followed by 40 cycles of 95 ℃ for 15 s, and 60 ℃ for 60 s. The cycle threshold (Ct) values were automatically calculated. The relative gene expression level was quantified using the 2^−ΔΔCt^ method and housekeeping gene *EF1-α* of *F. graminearum* was used as the reference [34]. All primers were designed using the online NCBI primers tool software (https://www.ncbi.nlm.nih.gov/tools/primer-blast, accessed on 20 May 2021) and listed in Table S1.

## 3. Results

### 3.1. Transcriptional Data Analysis of F. graminearum Infection on Soybean

In this study, the transcriptomes of infected soybean seedlings at three inoculated time points were sequenced using the Illumina HiSeq 2500 platform while the mycelia samples of *F. graminearum* were used as control. After removing low data quality including one sample at 24 hpi, expression data of 11 raw counts showed a maximum Pearson’s correlation among each biological repeat (>0.90), representing the high reproducibility of the sequencing data. As shown in Table 1, a total of 137.11 GB clean bases were achieved, and the value of GC content was more than 44.63% after filtering low-quality reads and adaptor sequences. Pair-end-clean reads ranged from 26,989,550 to 51,267,432 with average of 42,322,662. About 64.46 % of clean data were mapped to the *F. graminearum* genome, of which 56.91% were uniquely mapped reads (Table 1).

### 3.2. Differentially Expressed Genes (DEGs) of F. graminearum Infection on Soybean

**Figure 1:**
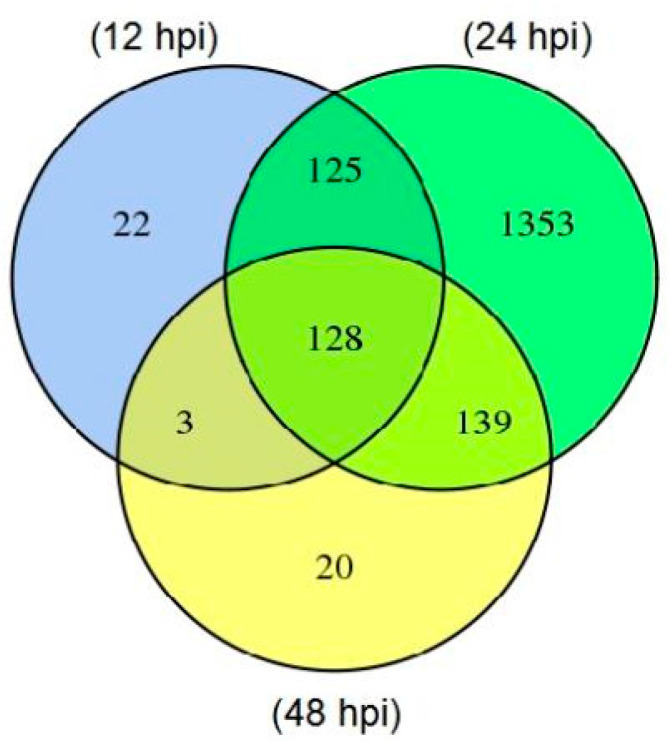
Differentially expressed genes (DEGs) at three time points after *F. graminearum* inoculation. The number of DEGs were calculated by comparison of samples at 12, 24 and 48 hpi versus the control *F. graminearum.*

**Figure 2:**
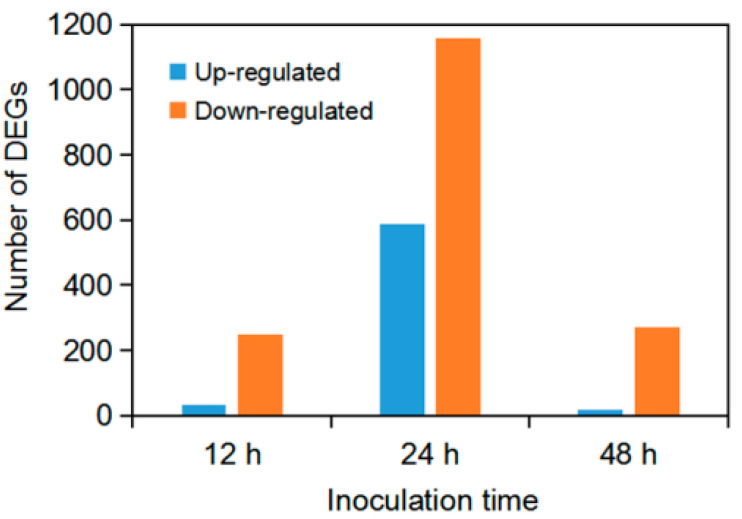
Number of up- and down-regulated DEGs at three time points after inoculation with *F. graminearum*.

After mapping genes of all samples with the *F. graminearum* reference genome from GenBank, genes from *F. graminearum* vs. samples at 12 hpi (T04_T05_T06), 24 hpi (T07_T09) and 48 hpi (T10_T11_T12) were defined as the differentially expressed genes (DEGs). A total of 2313 DEGs were annotated based on different functional databases, of which 278, 1745 and 290 genes were differentially expressed at 12, 24 and 48 hpi, respectively. DEGs were most frequently identified at 24 hpi while they were almost equal in numbers at 12 and 48 hpi. In addition, we found that 128 DEGs were commonly expressed at three indicated time points, but 22, 1353 and 20 DEGs were specifically expressed at 12, 24 and 48 h after *F. graminearum* inoculation, respectively (Figure 1). Furthermore, there were more down-regulated DEGs (1156/2313) than up-regulated ones (589/2313), and the maximum up- or down-regulated DEGs were appeared at 24 hpi (Figure 2).

### 3.3. DEGs Annotation of F. graminearum 

**Figure 3:**
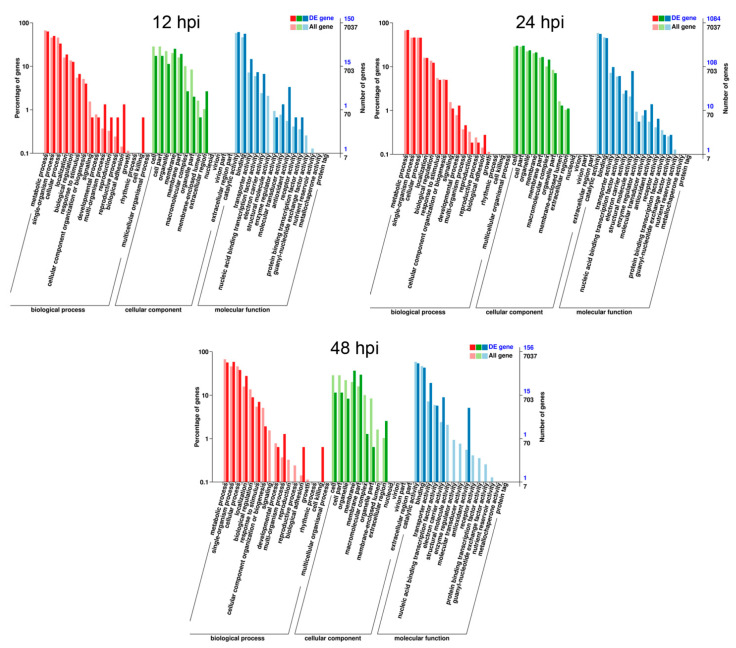
Gene ontology (GO) classifications of commonly induced genes at three time intervals after *F. graminearum* inoculation. Genes were annotated in three categories: cellular component (CC), molecular function (mf) and biological process (bp). All GO terms shown were significant at FDR ≤ 0.005.

To characterize the key genes involving in the infection and pathogenicity of *F. graminearum* on soybean, we predicted DEGs functions using GO terms and KEGG pathway. According to GO function terms, a total of 7037 genes were annotated into the cellular compartment, biological process and molecular functions, among which DEGs accounted for 4.9% (150/7037) at 12 hpi, 35.7% (1084/7037) at 24 hpi and 5.1% (156/7037) at 48 hpi, respectively (Figure 3 and Table S2). These DEGs of *F. graminearum* were significantly enriched in single organism process, metabolic process, cell, cellular process, biological regulations, localization, cell part, catalytic activity, extracellular regions, organelle, binding, nucleic acid binding transcription activity, transporter activity, and antioxidant activity (Figure 3 and Table S2). Among them, genes involved in the metabolic process, cellular process and single organism process were characterized by a high ratio at all three inoculation time points as mentioned in the Table S2. 

**Figure 4:**
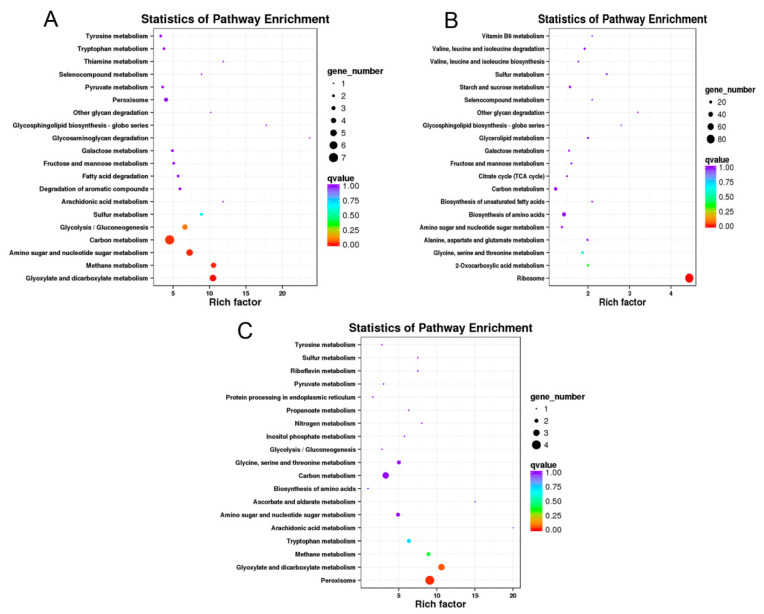
Top 19 KEGG pathway enrichment analysis of annotated DEGs after *F. graminearum* inoculation on soybean. Significantly enriched Encyclopedia of Genes and Genomes (KEGG) pathway of *F. graminearum* infection on soybean at 12 (**A)**, 24 (**B**) and 48 hpi (**C**). Solid dark circle showed the number of expressed genes and q value indicated the adjusted fold change value to <0.005.

For KEGG enrichment analysis, 27, 343 and 30 of DEGs from the total 1923 annotated genes were classified into 30, 92 and 19 KEGG pathways at 12, 24 and 48 hpi, respectively. The top 19 KEGG pathways at three time points were depicted as shown in Figure 4. The most KEGG pathways were annotated at 24 hpi (Table S3) and enriched into ribosome (84 DEGs), biosynthesis of amino acids (32 DEGs), carbon metabolism (24 DEGs), glycine, serine and threonine metabolism (16 DEGs) and other pathways involving in metabolic, genetic information processing and cellular process of *F. graminearum* (Figure 4B). Similarly, carbon metabolism and glyoxylate and dicarboxylate metabolism, peroxisome had relatively higher enrichment than other KEGG pathways at 12 and 48 hpi, respectively (Figure 4A,C). 

### 3.4. Validation of Randomly Selected Gene and Their Expression by qRT-PCR

**Figure 5:**
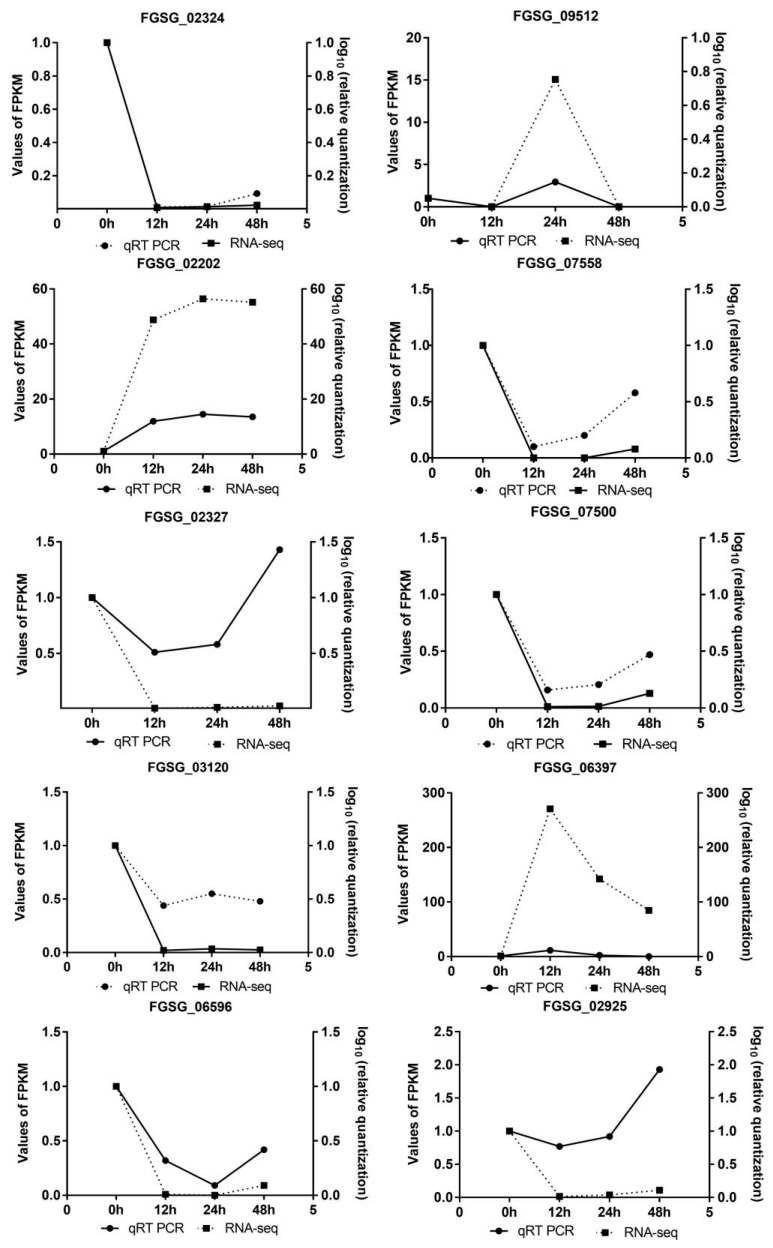
Expression change of 10 randomly selected genes of *F. graminearum* from RNA-seq. The expression pattern and level of selected genes from RNA-seq results were compared with those quantified by qRT-PCR. Quantitative analysis was conducted according to the normalization by the 2^−ΔΔCt^ method using the housekeeping gene *EF1-α* of the *Fusarium* genus.

In order to validate the gene expression data obtained from RNA-seq analysis, 10 genes of *F. graminearum* were randomly selected for qRT-PCR verification, and these genes were listed as *FGSG_02324*, *FGSG_09512*, *FGSG_02202*, *FGSG_07558*, *FGSG_07500*, *FGSG_02327*, *FGSG_03120*, *FGSG_06397*, *FGSG_06596*, *FGSG_02925*, involved in transport and catabolism, secondary metabolites biosynthesis, lipid transport and metabolism, amino acid transport and metabolism and some functionally unknown. The gene expressions were normalized by the 2^−ΔΔCt^ method using the housekeeping gene *EF1-α* of the *Fusarium* genus, and results showed that these selected genes have showed significant difference in relative quantity from RNA-seq analysis but had almost the same expression pattern in Figure 5. 

### 3.5. Expression Profile of DEGs Involving in Carbon Metabolism, Ribosome and Peroxisomes Pathway

As shown above, the expression profile of *F. graminearum* infection on soybean showed that carbon metabolism (7/27 DEGs), ribosome (84/343 DEGs) and peroxisome (4/30 DEGs) had the highest enrichment as compared to other pathways at 12, 24 and 48 hpi respectively (Figure 4 and Table S3). Among these pathways, peroxisome is very important because of its diversified function, and we found that 15 genes involving in the peroxisomes pathway of *F. graminearum* were up- or down-regulated upon its infection on soybean (Table 2). As shown in Figure 5, there was similar expression pattern of these genes verified by qRT-PCR and RNA-seq analysis. Among them, two genes (*FGSG_02881* and *FGSG_06596*) annotated as catalase (CAT), encoding one enzyme of detoxifying reactive oxygen species (ROS) in cells, were remarkably down-regulated when infected with soybean but the expression levels varied over infection time. Another gene (*FGSG_02051*) encoding Fe-Mn superoxide dismutase (SOD) also had significant down-regulated expression at 24 and 48 hpi. The gene *FGSG_00840* annotated as carnitine O-acetyltransferase was obviously down-regulated during the infection process with the log_2_FC value ranging from −2.58 to −3.30. In addition, two peroxin genes (*PEX2* and *PEX13*) were observed to be slightly down-regulated at 24 hpi. Interestingly, *FGSG_02287* and *FGSG_10347* mapped with acyl-CoA oxidase and isocitrate dehydrogenase were significantly up-regulated among all peroxisome pathway-related genes. These results indicate that these pathways have played their critical role in the infection and pathogenicity of *F. graminearum* at 12, 24 and 48 hpi when interacted with soybean.

## 4. Discussion

During the long-term interaction of plant and pathogen, a range of complicated and sophisticated signaling pathways regulated by pathogen genes have typically evolved to confront diverse plant hosts [35,36]. As one of the damaging agricultural pathogens, *F. graminearum* can infect a wide range of hosts, such as wheat, barley and maize as well as soybean, leading to diverse severe diseases [10,13,14,15,37]. The understanding of its molecular pathogenesis mechanism with a particular host provides valuable information for *F. graminearum*-related disease management. 

In the current study, we identified that 2312 DEGs of *F. graminearum* were differentially expressed in the infected soybean seedlings while 128 genes were simultaneously expressed at three infection time points. With regard to infection process, small amounts of DEGs were induced at early 12 hpi, whereas 1745 DEGs appeared at 24 hpi followed by a sudden decrease at 48 hpi, indicating the timeline at 24 hpi might be very critical for *F. graminearum* infection on soybean. However, expressed genes of *F. graminearum* increased sharply at 48 hpi but decreased at 144–96 hpi when infecting the host wheat [38]. Thus, when infected with different hosts, the infection process of *F. graminearum* can be significantly affected. Moreover, a study on cellular tracking of *F. graminearum* during maize stalk rot demonstrated that many genes encoding plant cell wall-degrading enzymes of *F. graminearum* were induced at 24 hpi and thus would probably be responsible for breaking the primary chains of pectin and causing inter- and intra-cellular hyphal growth in maize stalk [12]. Thus, an abundance of DEGs at 24 hpi can be predicted to function on intercellular hyphal growth, invasion and virulence. 

During the infection process, the phytopathogenic fungi were often exposed to oxidative stress caused by the oxidative burst, a rapid and transient accumulation of ROS as an early immune response in host plants [24,39]. These generated ROS can directly cause the damages of cell membranes by lipid peroxidation, DNA mutation, protein oxidation and even cell death [40]. Previous literatures demonstrated that necrotrophic fungi could employ a peroxisome pathway to balance ROS level and facilitate its colonization [41,42]. Other works showed that peroxisomes not only play vertical roles in detoxification of ROS and β-oxidation of fatty acids in fungi [43] but also contribute to biosynthesis of secondary metabolites, fungal penetration and virulence of *M. oryzae*, *A. alternata* and *F. graminearum* [27]. Several peroxin (PEX) proteins including PEX5, PEX6 and PEX7 have been identified to function on peroxisomal pathways and participate in ROS accumulation and necrotic cell death of host cells [44]. In our study, two genes peroxin 2 (PEX2) and peroxin 13 (PEX13) were significantly down-regulated, which might affect peroxisomal protein import and thus fail to detoxify ROS at the infection site. In *F. graminearum*, the spatial-temporal regulation of the antioxidant enzymes SOD and CAT contracts oxidative stress induced by host cells [42,45,46,47,48,49,50] and significantly reduces deoxynivalenol production leading to a weak virulence in wheat [51]. Our study showed that CAT and SOD encoding genes of F. graminearum were annotated in the peroxisomes pathways but significantly down-regulated, implying that their role in ROS scavenging might be inhibited by the immune signals of moderately resistant soybean cultivar Nandou12. Furthermore, we also found that genes encoding acetyl-CoA oxidase and isocitrate dehydrogenase involved in fatty acid β-oxidation [43,52] were remarkably up-regulated in *F. graminearum*, whereas the gene encoding peroxisomal carnitine O-acetyltransferase required for acetyl-CoA transport [53] and biosynthesis of trichothecenes and zearalenone [43,52] were significantly down-regulated, thus resulting in the reduction in mycotoxin biosynthesis as well as virulence of *F. graminearum*. 

Furthermore, our results demonstrated that ribosome and carbon metabolism have contributed their role in the invading of soybean upon inoculation. It was estimated that 7 DEGs of carbon metabolism (12 hpi) and 84 DEGs of ribosome (24 hpi) were regulated vigorously and facilitated the *F. graminearum*. Recently it was discovered that carbon metabolism and ribosome collectively responds in stress conditions and pathogenicity through the biosynthesis of sugar metabolism and secondary metabolism [54]. Except this, several other studies have also revealed that many genes encoding transcription factors, protein kinases and phosphatases involved in the DON synthesis and virulence of *F. graminearum* [55,56,57]. Accordingly, it can be concluded that genes in the ribosome and carbon metabolism pathways of *F. graminearum* were regulated negatively and positively when interacted with soybean which might affect mycotoxin biosynthesis and sexual reproduction, leading to the virulence of *F. graminearum* in soybean seedlings.

## 5. Conclusions

Conclusively, in the present study, we have identified a total of 2313 differentially expressed genes (DEGs) of a globally destructive pathogen *F. graminearum* after inoculation on soybean by RNA-seq analysis. Approximately 128 DEGs were commonly expressed at three different inoculation time points while the maximum DEGs were induced at 24 hpi. Functional and bioinformatics analysis implied that the carbon metabolism, peroxisomes and ribosome pathways in *F. graminearum* were up- or down-regulated in soybean infection, probably affecting ROS scavenging, mycotoxin biosynthesis, sexual reproduction and virulence of *F. graminearum.* Thus, it can be suggested that *F. graminearum* showed a stronger response from 24 hpi onward and explored the pathogenicity mechanisms and factors (pathways) that are involved in the *F. graminearum*-soybean interaction. However, further work is needed to interrogate the essential regulated genes of these three pathways of *F. graminearum* after inoculation on soybean.

## Figures and Tables

**Table 1:** Statically analysis of RNA-seq data.

Samples	Total Reads	Clean Reads	Mapped Reads	Unique Mapped Reads
12 h	53,979,100	26,989,550	283,660 (0.53%)	231,631 (0.43%)
12 h	82,859,646	41,429,823	393,276 (0.47%)	334,586 (0.40%)
12 h	100,333,768	50,166,884	399,024 (0.40%)	333,526 (0.33%)
24 h	79,795,198	39,897,599	3,202,980 (4.01%)	2,987,228 (3.74%)
24 h	72,608,382	36,304,191	3,455,298 (4.76%)	3,104,447 (4.28%)
48 h	88,036,166	44,018,083	6,783,005 (7.70%)	6,214,145 (7.06%)
48 h	72,301,850	36,150,925	6,423,924 (8.88%)	5,767,693 (7.98%)
48 h	74,999,418	37,499,709	5,774,290 (7.70%)	5,243,878 (6.99%)
*F. graminearum*	102,697,726	51,348,863	66,062,446 (64.33%)	58,398,509 (56.86%)
*F. graminearum*	101,416,397	50,176,223	64,173,223 (63.28%)	57,247,119 (56.48%)
*F. graminearum*	102,385,647	51,267,432	66,001,101 (64.46%)	58,266,178 (56.91%)

Notes: Samples, sample number of Baimaike analysis; Total reads, number of all single-end clean reads; Mapped reads, the number of the reads mapped to the reference genome and its percentage in total reads; Unique Mapped Reads: comparison of obtained total clean reads (%) and number of reads on the unique location of reference genome.

**Table 2:** Function and expression of genes of *F. graminearum* annotated in the peroxisomes pathway when infected on soybean seedlings.

Inoculation time	Gene ID	Description	log2FC
12 hpi	*FGSG_02881*	Catalase, CAT	Infinity
	*FGSG_06596*	Catalase, CAT	−5.82
	*FGSG_00840*	carnitine O-acetyltransferase	−3.18
24 hpi	*FGSG_00724*	peroxin-2, PEX2	−2.08
	*FGSG_00666*	peroxin-13, PEX13	−1.22
	*FGSG_02881*	Catalase, CAT	−7.33
	*FGSG_06596*	Catalase, CAT	Infinity
	*FGSG_02051*	superoxide dismutase (SOD), Fe-Mn family	−3.92
	*FGSG_02287*	acyl-CoA oxidase	2.46
	*FGSG_10347*	isocitrate dehydrogenase	2.06
	*FGSG_00840*	carnitine O-acetyltransferase	−2.580
48 hpi	*FGSG_02881*	Catalase, CAT	−2.28
	*FGSG_06596*	Catalase, CAT	−3.32
	*FGSG_02051*	superoxide dismutase (SOD), Fe-Mn family	−2.71
	*FGSG_00840*	carnitine O-acetyltransferase	−3.30

Note: log_2_FC means fold change in genes expression.

## Data Availability

The data will provide by the correspondence author on demand.

## Supplementary Materials

The following are available online at https://www.mdpi.com/article/10.3390/jof7060422/s1, Table S1: Primers used for the validation of selected genes in qRT-PCR. Table S2: Gene ontology (GO) enrichment analysis of Differently expressed genes (DEGs). Table S3: Kyoto Encyclopedia of genes and Genomes (KEGG) enrichment analysis of Differently expressed genes (DEGs).
