# Supplementary material for: Transcriptional Responses of Fusarium graminearum Interacted with Soybean to Cause Root Rot
Source: J Fungi (Basel). 2021 May 27;7(6):422. doi: 10.3390/jof7060422 (PMC8227214; doi:10.3390/jof7060422)
Supplement: Supplementary file 1 [file jof-07-00422-s001.zip › Supplementary material data/Supplementary material Table S1.pdf]

Supplementary material Table S1

Table S1: Primers used for the validation of selected genes in qRT PCR.

Primer Name	Primer sequence	Product length
FGSG_02324	TCAAGCTCCTCGGGTGACGA<br>TTTGGGCCCCATGCTGTTGT	252
FGSG_09512	AGGATGGCTGGAGCCCAAGA<br>CGAACTCGGCTGGCTCGAAA	215
FGSG_02202	AACACCGACAACGGCTTCGT<br>GCCTTGACCTTGGCGGACTT	101
FGSG_07558	AAGGGTTGCTGGCACTACGC<br>TGAAGCGGAGCATGCCAGAG	103
FGSG_02327	TCGACCGAGTGATTGGCTCT<br>CTGGCCAAGCCTTTCGCTTA	162
FGSG_07500	CCGTCATCCGCCAAGTCCTC<br>CGAGCGTCGTGTTTTCGCAC	233
FGSG_03120	ACGACACTCTTGCTGGTGCC<br>CAGCCAGCCTTGTGGGTGAA	181
FGSG_06397	TACTCTTTCCCCGGCCCCAA<br>GCCTCCTCGGAAGGCTTGAC	152
FGSG_06596	GCTACAACACGGCCAAGGG<br>GTCATCCGCAGCCTCAGCAA	167
FGSG_02925	CACGGTTCTCACCAGCAGCA<br>GTCCAACGGGGTCCTTGTGG	142
EF1-α	TGGCAAGTCGACCACTACCG<br>GGTGATACCACGCTCACGCT	163

Note: Primers were designed at NCBI for each gene and elongation translation factor gene (*EF1-α*) was used as reference primer for normalization in qRT-PCR.
